# Extensive Lateral Cervical Desmoid Tumor Dependent on the Levator Scapulae Muscle Involving the Vertebral Artery Treated by Surgical Resection and Intraoperative Navigation: A Case Report and Review of the Literature

**DOI:** 10.7759/cureus.63630

**Published:** 2024-07-01

**Authors:** Gonzalo Ruiz de León, Marta Benito-Anguita, Hubert Aranibar Meléndez, Carolina Agra Pujol, Saad Khayat

**Affiliations:** 1 Oral and Maxillofacial Surgery, Gregorio Marañón General University Hospital, Madrid, ESP; 2 Pathology, Gregorio Marañón General University Hospital, Madrid, ESP

**Keywords:** ctnnb1 mutation, intraoperative navigation, surgical resection, vertebral artery involvement, levator scapulae muscle, cervical mass, desmoid tumor

## Abstract

Desmoid tumors are rare, benign, but locally aggressive fibromatoses that pose significant therapeutic challenges, particularly when located in the head and neck region. This report details the case of an extensive cervical desmoid tumor dependent on the levator scapulae muscle and involving the vertebral artery managed through surgical resection and intraoperative navigation. A 45-year-old male presented with a slowly growing cervical mass. Imaging revealed an 83x68x40 mm mass in the right lateral paravertebral space, dependent on the levator scapulae muscle and involving the vertebral artery. Biopsy confirmed a low-grade fusocellular myofibroblastic neoplasm consistent with a desmoid tumor. Given the poor prognosis associated with the symptomatic mass, surgical resection was performed using Brainlab intraoperative navigation (Brainlab, Munich, Germany). The procedure was successful, with preservation of vital structures and no evidence of recurrence postoperatively. Desmoid tumors in the head and neck region, though rare, require precise diagnostic and therapeutic approaches due to their aggressive nature and proximity to critical anatomical structures. The use of intraoperative navigation, in this case, facilitated accurate tumor resection, minimizing damage to surrounding tissues. Pathological analysis revealed a CTNNB1 gene mutation, specifically the S45P variant, which is associated with an increased risk of recurrence. This case highlights the importance of a multidisciplinary approach, incorporating advanced surgical techniques and genetic analysis, in the management of complex desmoid tumors. Intraoperative navigation proved invaluable in achieving successful surgical outcomes, underscoring its potential utility in similar cases. Continued follow-up is essential, given the potential for recurrence associated with desmoid tumors.

## Introduction

Desmoid tumors are benign, non-metastasizing but aggressive deep-originating fibromatosis, first described by Muller in 1838 [1]. These tumors are not limited to any specific localization. They can be classified into intra-abdominal and extra-abdominal desmoid tumors, the latter being the most common group [2]. It’s a rare entity, with an incidence of approximately two to four cases per million/year [3]. Head and neck desmoid tumors are estimated to constitute up to 15% of all desmoid tumors in the body [3]. Their management in this region presents a therapeutic challenge, as they often grow invasively and may invade critical structures.

In this case report, we present the case of an extensive slow-growing cervical desmoid tumor dependent on the levator scapulae muscle and involving the vertebral artery, which was treated with surgery alone successfully, with satisfactory results and no evidence of recurrence.

## Case presentation

A 45-year-old patient with a history of dyslipidemia, autosomal dominant polycystic kidney disease, and arterial hypertension initially presented with an expansive cervical mass of slow progressive growth and indeterminate onset. He denied pain, although he reported mild limitation in head rotation. No coordination or balance abnormalities were reported, nor dyspnea or episodes of infection.

**Figure 1:**
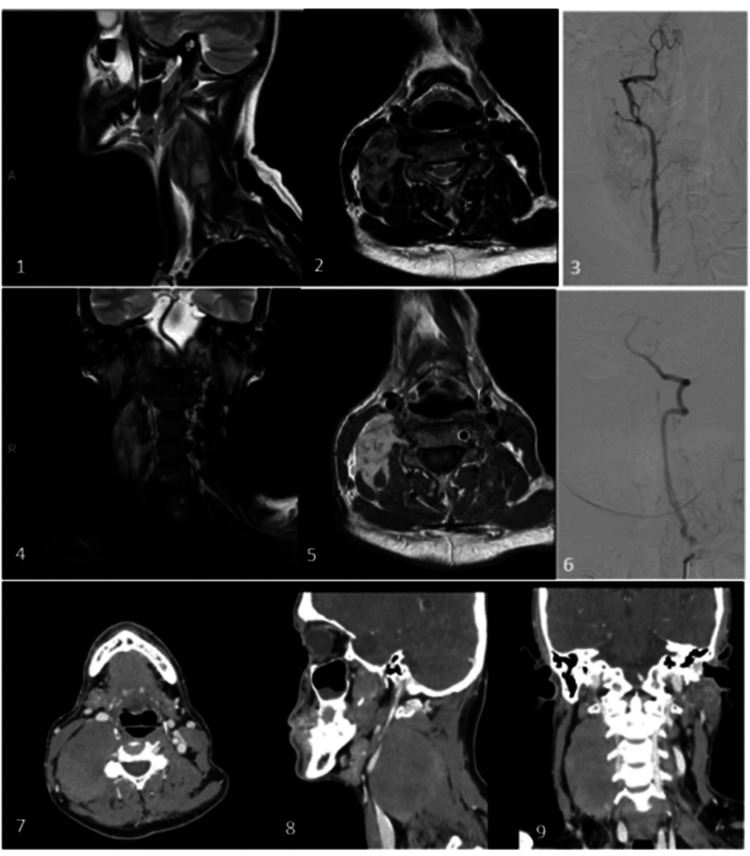
Patient's radiologic images Magnetic resonance imaging (MRI) shows a tumor next to the right levator scapulae, scalenus medius, and paravertebral muscles at levels C2 to C6 on the T2 sequence (1 and 2). It is closely related to the patient’s right vertebral artery (4) and presents an infiltrative pattern in the C3-4 and C4-5 foramina and adjacent muscles on the T1-weighted sequence (5). Vertebral artery angiography found segmental hypoplasia of the right vertebral artery after the posterior inferior cerebellar artery (PICA) branching (3) and significant atheromatous stenosis of the left vertebral artery at its origin (6), resulting in improbable compensation from the left side (negative right occlusion test). A CT scan revealed a tumor in the right paravertebral region (7) in contact with the internal jugular vein and no signs of infiltration (8); a cleavage space is maintained between the right sternocleidomastoid (SCM) muscle and the tumor (9).

Upon this finding, a biopsy was requested, reported as a low-grade fusocellular myofibroblastic mesenchymal neoplasm morphologically consistent with desmoid tumor. In addition, a contrast-enhanced cervical computed tomography (CT) scan was performed, revealing a right lateral paravertebral space mass, seemingly dependent on the levator scapulae muscle, with dimensions of 83x68x40 mm (Figure 1).

Its superior margin was approximately 15 mm above the plane of the inferior cortex of the mandibular angle. The caudal end was at the level of the superior horn of the thyroid gland. The anterior margin contacted the vascular space without infiltration, displacing and collapsing the internal jugular vein anteriorly. Good fat cleavage planes with the sternocleidomastoid muscle (SCM) were observed concerning the lateral margin. The medial and posterior margins showed organ-dependent changes of the levator scapulae muscle: loss of fat cleavage planes with the levator scapulae muscle superior to the C7 level, theoretically following its course to its insertion in transverse processes C1-C4 without evidence of bone infiltration. Regarding the vertebral artery, there was a loss of fat cleavage planes with the V2 intersomatic cranial segments to C4 and the beginning of the V3 segment, without conclusive infiltration. Contact without infiltration was noted with the insertion of the C4 middle/posterior scalene. No pathological cervical lymph nodes were observed.

Given the poor prognosis in a symptomatic patient, surgical treatment was decided. Prior to surgery, a right vertebral artery occlusion test was requested due to radiological evidence of its involvement. The test was uneventful, with a finding of significant stenosis in the origin of the left vertebral artery due to an atheromatous plaque, making catheterization impossible. 

**Figure 2:**
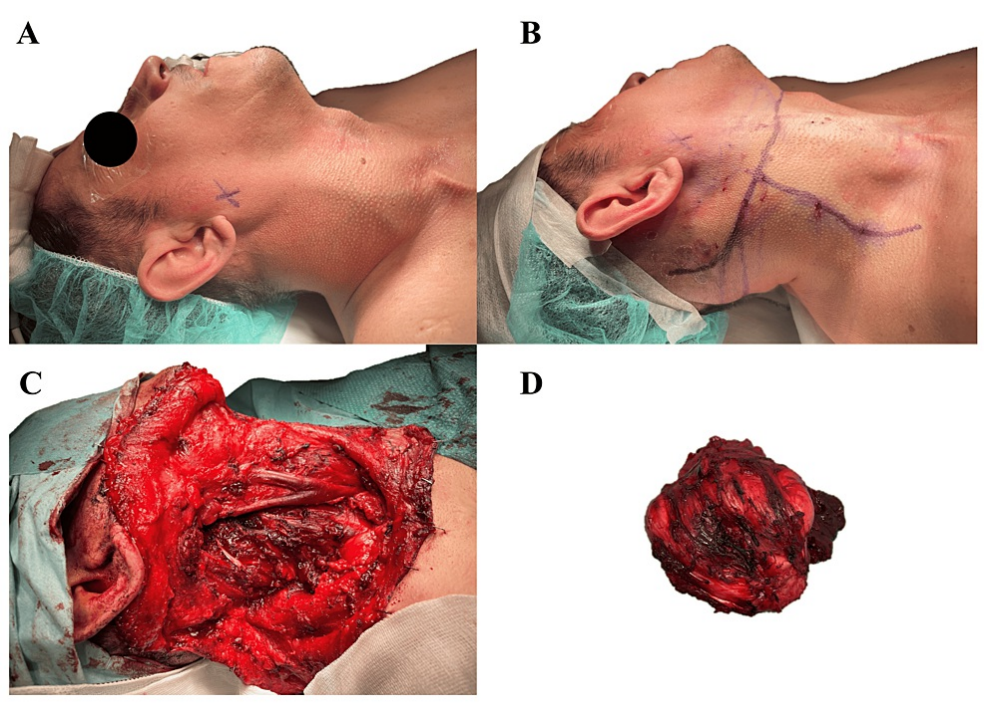
Intraoperative pictures A) Surgical site; B) Incisions are drawn over skin and infiltrated with local anesthetic and epinephrine; C) Surgical site after resection has been completed; D) Cervical tumor resected

Under general anesthesia and orotracheal intubation, Brainlab intraoperative navigation pins were placed on the left temporal area for eventual navigation. A right Schobinger approach cervicotomy was performed. Dissection was carried out through planes until the identification of the sternocleidomastoid muscle, which was detached at its clavicular portion. A whitish, stony, and rubbery-textured mass was identified and dissected, which revealed a completely collapsed internal jugular vein under the tumor. Preservation of the internal jugular vein was possible as it was dissected up to its cephalic portion. A posterior dissection of the tumor was performed, identifying the spinal nerve. Unfortunately, the spinal nerve was sacrificed due to its inclusion in the tumor. A good dissection plane between healthy tissues and the tumor was noted, allowing for a relatively easy and speedy dissection. Hemostasis was carefully performed on the vertebral venous plexus without damaging the right vertebral artery or cervical plexus. Phrenic and vagus nerves were identified and preserved. Resection was completed successfully, intraoperative navigation was performed to ensure surgical margins were attained, and two negative-pressure Redon drains were placed (Figure 2). 

During hospitalization, the patient maintained good pain control and did not report distal strength or sensory loss in the right upper limb. However, difficulty in shoulder abduction and elevation was noted. No coordination or balance alterations were observed. Drains were maintained for four days until their output was minimal. The patient was discharged after one week.

**Figure 3:**
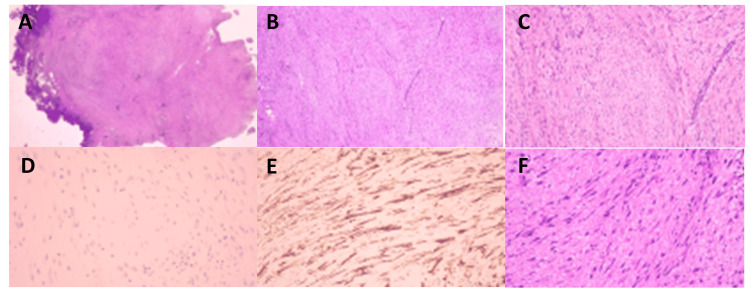
Final pathology Immunohistological analysis showed that there was positive nuclear staining for β-catenin. A mutation in the CTNNB1 gene was detected in exon 3, involving a T to C base change at nucleotide 133 (c.133T>C). This mutation resulted in the substitution of serine for proline at position 45 (p.S45P) in the protein product. A) (H&E, 2 times magnification) Low-magnification panoramic image of the tumor. A nodular spindle-cell formation with ill-defined borders, non-encapsulated in collagen stroma, is observed, contacting the circumferential surgical margin; B) (H&E 5X) Low-grade spindle-cell mesenchymal cellular proliferation with elongated poorly delimited eosinophilic cytoplasms and elongated nuclei, some with finely dispersed chromatin, generally isomorphic without cellular pleomorphism, growing in elongated parallel bundles within a hyaline collagen stroma with the presence of elongated curvilinear vessels; C) (H&E 20X) and D) (H&E 40X) Description similar to image B, with greater cellular detail, adding that a curvilinear vessel is observed on the right; E) Immunohistochemistry, IHC, 20X for Actin ML, Positivity of cells for Actin ML with focal cytoplasmic and nuclear staining is observed) F) (IHC 40X for Ki67) Nuclear positive staining in 1% of the cells is observed.

The final pathology revealed a low-grade myofibroblastic proliferation compatible with a desmoid tumor with a pathogenic variant in the CTNNB1 gene (Figure 3).

## Discussion

The desmoid tumor is an uncommon neoplasm in the head and neck region, representing 7-15% of all desmoid tumors in the body [3]. The neck is one of the most common sites, with the majority (80%) located in the anterolateral region, as in our case. It poses a challenge in both diagnosis and treatment due to the unique anatomy of this area. This type of tumor, despite lacking the potential to spread to other organs (metastasis), requires precise microscopic interpretation for correct identification, as it may present morphological similarities with other low-grade sarcomas [4].

Regarding potential etiological factors, the ethiopathogenesis is not fully understood. Up to 33% of patients with head and neck desmoid tumors reveal previous trauma or surgery at the tumor site [5]. In cases of sporadic desmoid fibromatosis (DF), there is a notable occurrence of mutations in the CTNNB1 gene. These mutations can cause unrestrained activation of the Wnt pathway, leading to the buildup of an excessive amount of cytoplasmic β-catenin. Ultimately, this accumulation contributes to the development of the tumor-associated with sporadic DF [6]. Moreover, tumors that are sporadic in nature are more commonly found in locations outside the abdominal area. Pregnancy and oral contraceptives have also been associated with the development of desmoid tumors. Intra-abdominal desmoid tumors have been reported more commonly in patients with Gardner syndrome or familial adenomatosis polyposis (FAP) [7].

In soft tissue tumors, fine needle biopsy is generally sufficient to establish an initial diagnosis to start treatment. However, the diagnostic output may be low, which would warrant the need for a core biopsy or an open one [8]. In this particular clinical case, initial morphological signs raised suspicion of a low-grade sarcoma, such as myxofibrosarcoma or malignant peripheral nerve sheath tumor. However, the absence of abnormal nuclear changes and the presence of smooth muscle actin, along with negative tests for certain markers (EMA, MUC4, and S100), allowed the exclusion of these more aggressive diagnoses. In our case, nuclear staining for β-catenin yielded a positive result. Immunohistochemical detection of β-catenin can be helpful in cases of desmoid tumors, which has a sensitivity between 82-100%. Despite this, several other entities also showed positivity for β-catenin nuclear staining: solitary fibrous tumors, endometrial stromal sarcomas, synovial sarcoma, fibrosarcoma, and clear cell sarcomas [9]. Numerous investigations also reported that using immunohistochemical methods to detect nuclear β-catenin did not prove to be a dependable means of differentiating between desmoid fibromatosis and benign or malignant fibroblastic lesions [10]. For this reason, CTNNB1 gene mutation analysis has been proposed as a more specific mean of diagnosis, and thus, was also performed. CTNNB1 mutation has been linked to a higher risk of recurrence [11].

There are three different types of mutations: T41A, S45F, and S45P. A recent analysis combining multiple studies revealed that the T41A and S45F mutations in the CTNNB1 gene are the prevailing mutations in desmoid-type fibromatosis. Among the 329 patients included in the analysis, T41A was identified in 154 patients (46.8%), and S45F was identified in 66 patients (20.1%). Additionally, the S45P mutation, while less frequent, was still notable, occurring in only 24 patients (7.3%) [12]. In another study performed by Penel et al. [13], the distribution of the three types of mutations was similar, the most common being the T41A variant (261 cases, 47%). Moreover, there was also an association between tumor size and p.S45F mutation; tumors harboring p.S45F tended to be larger. However, tumor size, rather than CTNNB1 mutation profile, was the main factor associated with event-free survival (EFS), as shown by their multivariate analysis. In our case, the main mutation revealed by immunohistochemistry was S45P. However, little information is available regarding the role of the CTNNB1 S45P mutation in cases of head and neck desmoid type fibromatosis due to few reported cases involving the S45P mutation in the diagnosis of DF.

Wide surgical resection with clear margins is the most common primary treatment for desmoid tumors. However, their location in the head and neck region can cause difficulties in complete removal due to their proximity to vital anatomical structures and their tendency for local recurrence (between 46% and 62%). Localizations near the brachial plexus, involvement of the deep cervical fascia, and tumors in the skin of the head were more likely to have positive surgical margins [2]. Despite the importance of obtaining clear margins, interventions prioritizing function and structure preservation of the affected area are preferred [2].

An important limitation in our case was the involvement of the vertebral artery (VA) and its management. In the context of addressing cervical vertebral column tumors, a critical component of the pre-operative surgical evaluation involves a comprehensive assessment of both the anatomical features of the vertebral artery and the extent of neoplastic involvement, as well as the eventual decision of whether or not to sacrifice it. In the algorithm proposed by Westbroek et al. [14], sacrificing the vertebral artery depends on three factors: tumor pathology, degree of VA encasement, and the redundancy of the patient's posterior circulation. In patients where an en bloc resection is feasible associated with a complete encasement (>180º) or invasion of the artery, a resection of the vessel is required. On the other hand, if the involvement is not complete, VA sacrifice should only be performed if the tumor capsule is at risk by skeletonizing the vessel. Preserving the artery is necessary if the contralateral circulation is not sufficient to support perfusion of the brainstem and cerebellum, as determined by angiography. Our case required us to preserve the VA as the contralateral vessel showed significant stenosis due to an atheromatous plaque, and the perfusion was likely to be compromised if the right VA was to be resected. 

A strong factor in the decision process that eventually led us to opt for surgical treatment was the use of intraoperative navigation. These systems provide real-time feedback during surgery, aiding in the precise localization of lesions and guiding tumor resection with minimal injury to adjacent tissues [15]. Navigation systems can reduce errors in specimen orientation, increase the distance of the tumor from resection margins, and improve overall oncological outcomes [16]. The technology allows for precise identification of bony landmarks, verification of tumor margins, and real-time tracking of deep references that are otherwise difficult to see if it weren't for the navigation. Additionally, navigation systems have been shown to reduce operative errors, improve surgical planning accuracy, and enhance patient safety during cervical tumor resections [15,16].

When managing desmoid tumors, the objective should be a wide (R0) microscopic margins resection, but in cases where positive (R1) microscopic margins are unavoidable due to functional or cosmetic concerns, they may be accepted. In instances where R1 resection is achieved in the initial management, there is insufficient evidence to recommend perioperative radiotherapy or re-operation [17]. Although combined modality appears to have a lower risk of local recurrence, the statistical significance between surgery alone and surgery plus perioperative radiotherapy is inconclusive. In situations where surgery is not a viable option and active management is necessary, moderate-dose definitive radiotherapy has demonstrated effective local control in a majority of progressive cases and can be considered, particularly when medical therapies are either unavailable or ineffective.

A "wait-and-see" strategy is recommended for certain patients with unresectable desmoid tumors or significant comorbidities. Given the unpredictable nature of the disease, patients require close monitoring. The decision to initiate active treatment should ultimately be guided by the patient's symptoms, overall health, and the biological characteristics of the tumor. [17]. Early active management can be considered if the disease is in proximity to a crucial structure, like mesenteric or head and neck desmoid tumors, due to the elevated possibility of substantial health challenges for the patient before achieving disease stabilization.

Radiotherapy carries a high risk of complications, particularly in the head and neck region, and should be reserved for patients who cannot undergo surgery due to significant comorbidities, in instances of residual or recurrent disease, or when surgery could substantially impair functional abilities. Chemotherapy and pharmacological treatments are options for unresectable tumors or when surgery and radiotherapy pose a high risk of significant morbidity. Medical treatment includes ani hormonal therapies such as tamoxifen or toremifene, combined with non-steroidal anti-inflammatory drugs (NSAIDs), tyrosine kinase inhibitors (imatinib, nilotinib, sorafenib, and pazopanib), and chemotherapy. Treatment with chemotherapy can involve a "low-dose" approach using a combination of methotrexate and vinblastine or vinorelbine. Alternatively, conventional chemotherapy may be employed, utilizing anthracycline-based regimens commonly used for treating soft tissue sarcomas, along with pegylated liposomal doxorubicin [17]. Despite different modalities of systemic treatments, there is no universal standard of care. A recently introduced secretase inhibitor has shown promising results in relation to progression-free survival and recurrences, with manageable adverse effects [18]. 

Recurrences in the head and neck typically occur within the first two years after initial surgery but can happen anytime from a few months to over ten years later [2,19]. MRI is the preferred imaging modality for investigating disease extension and conducting follow-up examinations due to its superior soft tissue definition compared to computed tomography. Because the natural behavior of these tumors is unpredictable, lifelong monitoring of patients is necessary [19].

## Conclusions

This case report illustrates the successful management of an extensive cervical desmoid tumor involving the levator scapulae muscle and the vertebral artery through surgical resection aided by intraoperative navigation. The complexity of the tumor's location required meticulous planning and execution to preserve vital structures while achieving complete resection. The utilization of advanced intraoperative navigation technology significantly contributed to the precision of the surgery, minimizing potential complications and ensuring clear surgical margins. Genetic analysis revealing a CTNNB1 mutation underscored the importance of molecular profiling in understanding tumor behavior and guiding postoperative monitoring. This case underscores the necessity of a multidisciplinary approach in treating head and neck desmoid tumors, combining surgical expertise, technological innovation, and genetic insights to optimize patient outcomes. Continuous follow-up is essential due to the risk of recurrence associated with desmoid tumors.
